# Feasibility of ApoC1 serum levels as tumor biomarker in glioblastoma patients: a pilot study

**DOI:** 10.1038/s41598-022-21216-1

**Published:** 2022-10-10

**Authors:** Michelle Hilbert, Peter Kuzman, Wolf C. Mueller, Jürgen Meixensberger, Ulf Nestler

**Affiliations:** 1 Department of Neurosurgery, University Hospital Leipzig, Liebigstrasse 20, 04103 Leipzig, Germany; 2 Paul-Flechsig-Institute of Neuropathology, University Hospital Leipzig, Leipzig, Germany

**Keywords:** CNS cancer, Lipoproteins, Predictive markers

## Abstract

Apolipoprotein C1 (ApoC1) has been detected immunohistochemically in glioblastoma tissue, probably expressed by activated monocytes and microglia. The present study was conceived to determine whether the amount of intratumoral ApoC1 expression leads to measurable changes of serum levels after glioblastoma resection or during recurrence. 176 blood samples from 70 glioblastoma patients were collected perioperatively and during subsequent therapy. ApoC1 serum levels were determined using an enzyme linked immunosorbent assay (ELISA). High absorption values due to lipemic or hemolytic serum were removed from the final dataset using a stem and leaf plot. Samples were grouped according to the treatment stage to compare mean ApoC1 serum levels. The number of patients with falling or increasing perioperative values was assessed. 167 ApoC1 serum values from 68 glioblastoma patients were amenable to statistical evaluation. Mean ApoC1 serum level was 91.9 µg/ml (n = 167, sd = 36.0). In samples from patients undergoing first glioblastoma resection, the mean preoperative value was significantly higher (94.8 µg/ml, n = 37, sd = 29.5) than after surgery (77.4 µg/ml, n = 41, sd = 23.2, *p* = 0.009). Individually, falling ApoC1 levels were detected in 25 and rising levels in 9 patients (*p* = 0.0061). Single absolute serum levels of ApoC1 do not allow an estimation of glioblastoma activity or tumor response. Although pathophysiologically of interest, ApoC1 serum levels did not qualify as a potential biomarker in glioblastoma management. Our results do not seem to encourage larger, multicenter studies.

## Introduction

Mass spectrometric analysis has shown the presence of apolipoprotein C1 (ApoC1) in glioblastoma cyst fluid^1^. This finding was confirmed in solid glioblastoma tissue by assessment of mRNA expression and by immunohistochemical staining^2^.

ApoC1 is mainly synthesized by liver cells to be integrated into high density lipoproteins and exerts a blocking function on low density lipoprotein receptors. The protein is coded on chromosome 19q13.32 and is expressed in several splice variants, mainly with a size of 6631 Dalton^1^. Astrocyte-based expression of ApoC1 in the central nervous system has been described^3^. The ApoC1 gene is expressed when monocytes differentiate into macrophages.

Suppression of toll-like receptor dependent activation of microglia by ApoC1 has been observed, hypothetically constituting an autocrine loop for modulation of immune response or even enabling an immune escape of glioblastoma cells^3^. Consequently, higher ApoC1 expression could correspond to larger glioblastoma volume or recurrent growth of tumor cells. On the other hand, intratumoral necrosis is a histologic feature of malignant glioma and involves large amounts of phagocytic cells. Since up to 40% of glioblastoma tissue consist of activated monocytes or microglia, detectable amounts of ApoC1 in glioblastoma samples can occur^4,5^. From this point of view, ApoC1 expression would mirror the activity of tissue macrophages.

Theoretically, due to the tumor-induced damage of the blood brain barrier, and after neurosurgically provoked local disruption, ApoC1 may be swept into the blood stream and consecutively lead to measurable changes of ApoC1 serum levels. Accordingly, a first assessment in neurosurgical patients had disclosed a significant drop of ApoC1 serum levels after the surgical intervention^6^. Here we present a detailed analysis of serum samples exclusively from glioblastoma patients, examined using a commercially available enzyme linked immunosorbent assay (ELISA). A correlation of these values to tumor volume or the clinical course of the patients would then eventually allow the use of ApoC1 as serum biomarker for glioblastoma.

## Patients and methods

The study protocol, procedures and patient informed consent form had been approved by the local ethics committee prior to the study (311/19-ek, Medizinische Fakultät Ethik-Kommission Leipzig). 176 serum samples from 70 glioblastoma patients were collected prospectively before and after neurosurgical interventions, as well as from patients presenting to the outpatient clinic during follow-up visits for chemotherapy.

Follow-up visits were routinely scheduled in 3-months intervals with the neurosurgical intervention as starting point. The routine serum samples for leukocyte and thrombocyte counts during chemotherapy were taken both during concomitant chemotherapy or during maintenance cycles. According to the patients course, the sampling time points varied from 1 to 227 months postoperatively (mean 18.7 months, median 10 months).

Prospectively collected samples were frozen at – 20 °C until assessment by enzyme linked immunosorbent assay (EHAPOC1, Invitrogen, Waltham, MA) according to the instructions of the manufacturer. Samples were assessed in duplicate and in two dilutions (1:10.000 and 1:50.000). The 96-well plate was read twice in a photometric microplate reader and control absorption at 550 nm was subtracted from the 450 nm absorption values. The 8 obtained values were averaged and expressed in µg/ml.

Demographic data of the patients were extracted from the digitally archived records. To assess a potential influence of liver function or perioperative blood loss on ApoC1 concentration, serum values for ALAT, ASAT, bilirubin, C-reactive protein, hemoglobin and leukocyte count were obtained from the sample or the same day as the ApoC1-sample. In order to examine the ApoC1 correlation to tumor burden, MRI tumor volume [ml] on imaging corresponding to the date of serum withdrawal was calculated. The formula of a rotational ellipsoid (0.52 × A × B × C) with assessment of the three diameters A, B and C [cm] in axial, sagittal and coronal T1 weighted MRI after application of contrast medium was employed.

Evaluation of data was performed using SPSS 27 (IBM, Armonk, NY). Outliers and extreme values due to lipemic or hemorrhagic contamination of ApoC1 samples were identified by stem-and-leaf plot and removed. Statistical significant differences for mean values were assessed by Mann–Whitney-U test and for categorical variables by chi-square test. Parameters with significant differences between preoperative and postoperative mean values were correlated to the ApoC1 serum levels based on individual samples, and diagnosis of proportionality was assessed by linear regression.

### Ethical approval

The collection of specimens, data acquisition and experimental procedures had been approved by the local ethics committee (311/19-ek, Ethik-Kommission der Medizinischen Fakultät der Universität Leipzig) and were performed in accordance with the ethical standards of the Declaration of Helsinki and its later amendments. All patients have given informed consent prior to inclusion into the study.

## Results

After removal of 9 lipemic/hemorrhagic samples with ApoC1 extreme values over 227 µg/ml according to the SPSS-based stem-and-leaf plot, the remaining dataset comprised 167 samples from 68 patients with a mean age of 64.06 years and an even distribution between men and women (34 each). 37 samples were obtained before the first neurosurgical intervention for glioblastoma, 8 before a second, and 3 before a third surgery. 67 specimens were sampled during chemotherapy. Histologically, in 64 patients the tumor was diagnosed as glioblastoma IDH-wild type, in one as gliosarcoma IDH-wild type, and in three glioblastomas an IDH mutation was disclosed. Histologic assessment refers to the 2016 revision of the 4th edition of the WHO classification of central nervous system tumors, since sampling of tumor specimens ended in December 2020.

There was no statistical difference in mean ApoC1 serum levels, neither concerning patient characteristics as gender, age or months after neurosurgical resection, nor in view of ELISA characteristics such as timing of the test after the start of the study or seasonal influences.

Concerning the first neurosurgical intervention for glioblastoma, the mean preoperative ApoC1 serum level was significantly higher than the mean postoperative value (Table 1). Similarly, the mean postoperative ApoC1 serum level was significantly lower than the level during chemotherapy. These findings were confirmed when additionally including patient samples from second or third interventions. Also, on an individual patient base, significantly more patients had lower ApoC1 serum values after surgery than before.

**Table 1:** Mean ApoC1 levels and changes in individual patients after surgery for glioblastoma.

ApoC1 serum values in glioblastoma patients			
Samples	Number	Mean [µg/ml]	SD
Whole group	167	91.9	36.0
Preoperatively (first neurosurgery)	37	94.8^a^	29.5
Postoperatively (first neurosurgery)	41	77.4^a,b^	23.2
Preoperatively (all)	48	95.2^c^	33.7
Postoperatively (all)	52	78.3^c,d^	23.4
During chemotherapy	67	100.1^b,d^	42.5

Individual ApoC1 serum values after neurosurgery			
Patient number	Falling	Increasing	Chi-square p
First neurosurgery (n = 34)	25	9	0.0061
All (n = 45)	33	12	0.0010

^a,b,c,d^Values with the same superscript letter differ significantly (*p* < 0.05 Mann–Whitney-U test).

SD = standard deviation.

First neurosurgery = only patients undergoing their first intervention for glioblastoma.

All = including patients with first, second or third intervention for glioblastoma.

When assessing the potential confounding factors, mean postoperative values were found to be significantly lower for MRI tumor volume and hemoglobin. In contrast, serum levels for bilirubin and CRP were rising (Table 2). There were no statistically significant perioperative differences concerning ALAT, ASAT or leukocyte count. Of note, when assessing the individual patient serum pairs by linear regression, no proportionality between ApoC1 changes and the four other parameters was detected (Table 2).

**Table 2:** Perioperative changes in ApoC1 level, tumor volume, bilirubin, CRP and hemoglobin.

Parameters with significant differences between preoperative and postoperative values after first neurosurgical intervention					
Mean values	ApoC1	MRI tumor volume	Bilirubin	CRP	Hemoglobin
	[µg/ml]	[ml]	[µmol/l]	[mg/l]	[mmol/l]
Normal range	See Table 3	0	< 17.1	< 5	7.5–9.9
Preoperative	94.8	35.5	7.7	3.2	8.7
Postoperative	77.4 *	11.8*	10.2*	22.2*	7.6 *
Change in %	− 18.4	− 66.8	32.5	593.8	− 12.6
**Patient number (n = 34)**					
Falling	25	34	6	2	33
Increasing	9^#^	0^#^	26^#^	17^#^	1^#^
Missing			1	15	
Unchanged			1		
Potential confounder		Resection	Anesthesia-induced	Post-aggression metabolism	Dilution effect
Correlation to ApoC1 (linear regression)		No	No	No	No

*p < 0.05 Mann–Whitney-U test,

^#^*p* < 0.05 chi-square test.

**Table 3:** Overview of published ApoC1 serum levels.

ApoC1 serum levels in different populations				
Year	Author	Population	Patients	Level [µg/ml]
1981	Curry et al^7^	Control	n.d	60.0
		Hyperlipoproteinemia	n.d	132.5
1982	Carlson and Holmquist^8^	Normolipidemic men	29	63.0
1986	Riesen and Sturzenegger^9^	Apparently healthy males	38	61.0
		Apparently healthy females	32	65.0
1987	Attman et al^10^	Control	42	85.5
		Chronic renal failure	33	93.0
1993	Bren et al^11^	control	26	72.0
		Diabetes	14	113.2
		Type V hyperlipoproteinemia	12	137.8
2003	Cohn et al^12^	Normolipidemic	89	105.7
		Hyperlipidemic	88	128.7
2005	Shachter et al^13^	Hispanic children	362	62.0
2007	Dautin et al^14^	Chronic renal failure	28	131.1
2008	Berbée et al^15^	Sepsis	17	13.4
2010	Lahiry^16^	Male Oji-Cree	192	220.7
		Female Oji-Cree	217	203.9
	Xue et al^17^	Control	18	107,000.0
		Pancreatico-biliary pathology	28	101,000.0
2011	Cohen et al^18^	Control	54	92.5
2013	McNeal et al^19^	Vascular	20	115.4
2014	Ko et al^20^	Control	8	50.0
		Pneumonia	16	50.0
2018	Al-Daghri et al^21^	Vitamin D substitution	120	35.2
	Dittrich et al^22^	control	1300	40.0
2019	Wang et al^23^	Control	60	17,390.0
		Chronic atrophic gastritis	60	12,830.0
	Yi et al^24^	Control	40	0.1
*Non-tumor patients*	*Number*	2943		
		*Mean*		8584.0
		***Median***		**92.8**
2010	Xue et al^17^	Pancreatic adenocarcinoma	33	124,000.0
2011	Cohen et al^18^	Stomach cancer	101	31.3
2014	Ko et al^20^	Lung cancer	48	50.0
2019	Wang et al^23^	Gastric cancer	60	9530.0
	Yi et al^24^	Gastric cancer	65	0.3
	Present study	glioblastoma	68	91.9
*Tumor patients*	*Number*	375		
		*Mean*		22,283.9
		***Median***		**71.0**

n.d.: not disclosed. Median values are in bold.

**Figure 1:**
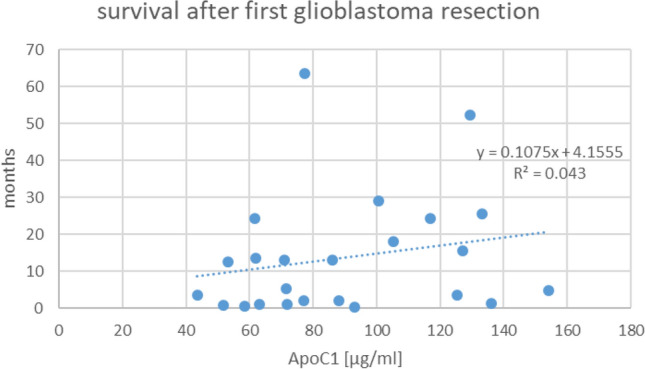
Survival time of 24 patients in function of their averaged ApoC1 serum levels (mean value from all samples taken in an individual patient during the study period). The deviation of the regression line from horizontal is not significant.

During the follow-up period, 24 patients succumbed to their disease. In the 58 according samples, the mean ApoC1 level had been 93.79 µg/ml (SD 37.69), not differing from the mean level in the study group. The relationship of ApoC1 levels to survival time was then assessed by linear regression analysis. Neither relating single absolute ApoC1 values to remaining life span, nor relating averaged values per patient to survival time from first surgery, showed a significant correlation (Fig. 1).

In 33 patients receiving chemotherapy, multiple sequential ApoC1 levels were available. Only in 5 (15.2%) of these patients the last obtained sample during follow-up showed the highest ApoC1 level, no correlation to treatment stage or clinical course was disclosed.

In another subset of 33 patients, tissue slices were assessed immunohistochemically for ApoC1 expression, using a semiquantitative scale^2^. Only one slide was scored as negative, the corresponding serum sample showed an ApoC1 value of 87.06 µg/ml. The highest staining score was also found only once, with a corresponding serum value of 64.84 µg/ml. Low staining scores were attributed in 8 cases, medium scores in 18 and high scores in 5 slides. A significant correlation of the immunohistochemical ApoC1 expression to serum levels or to survival time was not found.

## Discussion

In the present cohort, a significant decrease of mean ApoC1 serum levels was detected after glioblastoma resection. In accordance with this, significantly more patients had falling ApoC1 serum values after neurosurgery than rising values.

A correlation to confounding medical factors, such as reduction of tumor volume, anesthesia induced changes in liver function, dilution effects or post-aggression metabolism was excluded.

However, the lack of correlation of ApoC1 serum values to tumor volume on MRI and to protein expression in histological slides, as well as the missing relationship of ApoC1 levels to survival time do not favor the use of ApoC1 as serum biomarker or prognostic marker for glioblastoma.

Our results in glioblastoma patients mirror the ongoing discussion in current publications concerning malignant diseases (Table 3). Two studies dealing with gastric cancer found higher ApoC1 serum levels to be associated with a better prognosis^18,23^. In contrast, another recent publication described the tumor burden in gastric cancer to be elevated in the presence of higher ApoC1 levels^24^. An analysis in pancreatic adenocarcinoma stated that ApoC1 serum levels add to the diagnostic accuracy as part of a biomarker panel^17^. An examination of lung cancer patients led to the conclusion, that the use of ApoC1 serum values does not fulfill the criteria for a biomarker^20^.

Similarly, the overexpression of ApoC1 mRNA has been analyzed in a multitude of malignancies, especially renal cancer and colorectal cancer, but also gynecological tumors, prostate cancer and pancreatic or gastric carcinoma. The conclusions remain equivocal, whether an elevated mRNA expression is associated with a better prognosis of the patient. Of note, a high mRNA expression can occur without a subsequently elevated protein synthesis, the cellular subpopulations of the tumor that shed ApoC1 into the blood stream being subject to further translational control.

According to the results of the present study, namely a poor correlation of ApoC1 serum levels to tumor volume and to immunohistologic staining intensity, the subset of neuroepithelial cells does not constitute an important source of circulating ApoC1 protein. The increased detection of ApoC1 immunostaining in the vicinity of necrotic areas favors the theory of its occurrence during the activation of microglia and invading macrophages^2,3^. In this way, expression of ApoC1 can be considered to be a physiological reaction of surrounding tissue and immunocompetent cells to the stimulus by the glioblastoma cells, similar to stromal reactions described in other malignant entities, and in line with increasing ApoC1 serum levels detected during recovery from sepsis^15,25^.

In the cohort presented here, the stimulus and its removal were sufficient to result in detectable and significant serologic ApoC1 level changes. When comparing these values to the results from the literature, it remains questionable whether the glioblastoma-derived ApoC1 synthesis overrides the levels obtained by physiologic production in the liver or postprandial hyperlipoproteinemia (Table 3). Thus, a single absolute ApoC1 serum value does not allow conclusions concerning the activity of the glioblastoma.

As a next step, to determine whether monitoring of subsequent serum levels leads to the detection of a recurrent tumor before it becomes visible in MRI, a much larger, multicenter study with a long-time follow-up would be needed. The patients should preferentially undergo strict fasting before drawing the serum and the samples should be assessed with a high-throughput technique such as liquid chromatography mass spectrometry, allowing for the simultaneous detection of further apolipoproteins^22^. In order to exclude hepatic, vascular or obesity-induced interferences, a normalization of values to body weight or hepatic volume might be undertaken. These settings and prerequisites then considerably interfere with the ideal of a simple and easy to use biomarker.

## Conclusion

In the examined patients, ApoC1 serum levels fell significantly after neurosurgical resection of glioblastoma. However, no cut-off values to define tumor control or recurrence could be detected. Repeated monitoring of ApoC1 levels in a large number of individual patients, recruited in a multicenter study would be needed, to determine whether ApoC1 correlates to or is predictive for clinical symptoms and MR-imaging.

The present study yields novel and pathophysiologically most interesting information about protein shedding from glioblastoma tissue, even giving a hint on how local tumor entities might influence the systemic lipid metabolism of the host. From a practitioner’s point of view, the use of serum ApoC1 levels as glioblastoma biomarker with an impact on clinical or diagnostic decision-making seems out of reach.

## Data Availability

The datasets generated and analyzed during the current study are not publicly available due to technical reasons but are available from the corresponding author on reasonable request.

## Abbreviations

ALAT: Alanine-aminotransferase
ASAT: Aspartate-aminotransferase
ApoC1: Apolipoprotein C1
CRP: C-reactive protein
ELISA: Enzyme linked immunosorbent assay
IDH: Isocitrate dehydrogenase
µg/ml: Microgram per milliliter
mRNA: Messenger ribonucleic acid
MRI: Magnetic resonance imaging
n: Number
sd: Standard deviation
